# Single-cell ATAC sequencing identifies sleepy macrophages during reciprocity of cytokines in *L. major* infection

**DOI:** 10.1128/spectrum.03478-23

**Published:** 2024-02-01

**Authors:** Shweta Khandibharad, Shailza Singh

**Affiliations:** 1 Systems Medicine Lab, National Centre for Cell Science, SP Pune University Campus, Pune, India; Hebrew University of Jerusalem, Jerusalem, Israel

**Keywords:** single-cell ATAC sequencing, sleepy macrophages, parasite, systems biology, transcription factor

## Abstract

The hallmark characteristic of macrophages lies in their inherent plasticity, allowing them to adapt to dynamic microenvironments. *Leishmania* strategically modulates the phenotypic plasticity of macrophages, creating a favorable environment for intracellular survival and persistent infection through regulatory cytokine such as interleukin (IL)-10. Nevertheless, these effector cells can counteract infection by modulating crucial cytokines like IL-12 and key components involved in its production. Using sophisticated tool of single-cell assay for transposase accessible chromatin (ATAC) sequencing, we systematically examined the regulatory axis of IL-10 and IL-12 in a time-dependent manner during *Leishmania major* infection in macrophages Our analysis revealed the cellular heterogeneity post-infection with the regulators of IL-10 and IL-12, unveiling a reciprocal relationship between these cytokines. Notably, our significant findings highlighted the presence of sleepy macrophages and their pivotal role in mediating reciprocity between IL-10 and IL-12. To summarize, the roles of cytokine expression, transcription factors, cell cycle, and epigenetics of host cell machinery were vital in identification of sleepy macrophages, which is a transient state where transcription factors controlled the epigenetic remodeling and expression of genes involved in pro-inflammatory cytokine expression and recruitment of immune cells.

**IMPORTANCE:**

Leishmaniasis is an endemic affecting 99 countries and territories globally, as outlined in the 2022 World Health Organization report. The disease’s severity is compounded by compromised host immune systems, emphasizing the pivotal role of the interplay between parasite and host immune factors in disease regulation. In instances of cutaneous leishmaniasis induced by *L. major*, macrophages function as sentinel cells. Our findings indicate that the plasticity and phenotype of macrophages can be modulated to express a cytokine profile involving IL-10 and IL-12, mediated by the regulation of transcription factors and their target genes post*-L. major* infection in macrophages. Employing sophisticated methodologies such as single-cell ATAC sequencing and computational genomics, we have identified a distinctive subset of macrophages termed “sleepy macrophages.” These macrophages exhibit downregulated housekeeping genes while expressing a unique set of variable features. This data set constitutes a valuable resource for comprehending the intricate host-parasite interplay during *L. major* infection.

## INTRODUCTION

Effective immune functioning is influenced over the course of a lifetime by a niche of foreign entities through anthroponotic or zoonotic transfer. For combating these infections, immunological defense systems trigger phagocytosis or apoptosis, produce cytokines or antibodies, and release inflammatory or cytotoxic mediators (1). Nonetheless, the impact of infectious diseases is still substantial in low- and lower-middle-income countries, and the mortality and morbidity attributable to neglected tropical diseases are also high (2), of which leishmaniasis is believed to have a high mortality rate in humans and is further correlated to the disadvantages of current medications, such as their high toxicity and drug resistance (3). Leishmaniasis is an intracellular protozoal infection that is chronic and is caused by multiple species of the genus *Leishmania*. Lines of evidence point toward leishmaniasis becoming more common due to factors like climate change, the discovery of effective vectors and reservoirs, a highly mobile population, significant population groups with documented exposure histories, HIV, and the widespread use of immunosuppressive drugs and organ transplants, encouraging potential for sustained autochthonous spread (4). Apart from standard drugs and Food and Drug Administration-approved drugs such as pentavalent antimonials, meglumine antimoniate, sodium stibogluconate, pentamidine, liposomal amphotericin B, and miltefosine, (3) novel therapeutic approaches are also progressing and gaining a lot of attention, such as phytotherapy, nanotherapeutics, metabolite encrichment, anti-microbial peptides, proteasomes, and epigenetic modifiers (5).

The chromatin remodeling in the parasite is thought to be significantly influenced by altered epigenetic histone modifications. It is intriguing to note that the parasite alters host gene expression as well, enabling the host immune response to be suppressed or hijacked. The silencing of genes specific to macrophages that are involved in defense against these parasites has been linked to epigenetic factors such DNA methylation of cytosine residues (6). Many studies highlight the potential of epigenetic factors as a prime target for vaccine as well as therapeutic target development. Recombinant histone H1 vaccination of monkeys resulted in a slower development of cutaneous lesions compared to controls, suggesting histone H1 can be a candidate for vaccine development against cutaneous leishmaniasis (CL) in humans (7). It was also reported that rLdH2-4 exclusively generates Thl-type immune responses, providing significant protection against experimental visceral leishmaniasis that cured patients/endemic contacts and hamsters (8). It was also shown that cutaneous lesions exhibit amplified matrix metalloproteinase 1, which may be regulated epigenetically by Friend leukemia virus integration 1 (FLI1) and interleukin (IL)-6, suggesting FLI1 can be a therapeutic target (9). Promising therapeutic targets might include the enzymes responsible for histone post-translational modifications, particularly those that contain epigenetic reader modules and bromodomains such as sirtuins of *Leishmania donovani* (6). The effect of imipramine on IL-10/IL-12 axis reciprocity was studied for clearance of antimony-resistant *L. donovani*, which specifically targeted host HDAC11, which inhibited its ability to acetylate IL-10 promoter region, leading to downregulation of IL-10 (10). Even though these vaccines and drugs with potentials to inhibit parasite growth have been reported, none of them has been introduced in the market specifically targeted toward any form of leishmaniasis. The shortcomings and limitations of these molecules are the phenotypic response of immune cells. In order to understand drug design, it is important to understand immune cell population as a network and dissecting the time-dependent response of the host immune cell toward parasite. Therefore, population dynamics, cell enrichment, phenotype characterization, and heterogeneity distribution may lay an insight as to which subsets of cells are playing a role in parasite proliferation and which genes are supporting the parasite survival process.

Majority of single-cell profiling investigations have so far relied on quantifying RNA by sequencing (single-cell RNA sequencing). While this offers glimpses of the intercellular and intracellular variations in gene expression, investigation into the epigenomic landscape in single cells has enormous promise for revealing a key component of the regulatory logic of gene expression programs (11). Together with the help of recent developments in array-based technologies, droplet microfluidics, and combinatorial indexing through split-pooling, the single-cell assay for transposase accessible chromatin using sequencing (scATAC-seq) has been able to generate chromatin accessibility data for thousands of single cells in a relatively simple and affordable way (11). Conventional methods that use bulk tissue samples as input lack the resolution to assess the temporal dynamics of cell type-specific use. This methodology has been successfully implemented to embryonic tissues in *Drosophila melanogaster*, developing mouse forebrains, adult mouse tissues (12), human pancreatic islets (13), human testicular cells (14), fetal human retina (15), and mouse cardiac progenitor cells (16). This technique enables separation of cells by discriminating them on the basis of cell types, source, and cell variability to generate population clusters (17).

scATAC-seq is based on bulk ATAC-seq, which involves isolation of nuclei from single cells in the sample through fluorescence-activated cell sorter (FACS). Hyperactive Tn5 transposase catalyses the process of tagmentation by integrating sequencing adaptors to the targeted DNA by initiating its binding to the DNA followed by release from DNA post-tagging through heat or denaturing molecules. While nuclei are still intact, single nuclei are isolated (18). The nuclei are later lysed and loaded onto 96-well plates containing specially barcoded transposases, then sorted again before being dispensed into a second 96-well plate for FACS. Later, a second set of barcodes known as unique molecular identifiers are introduced in the amplification step (19). By identifying a distinct combination of both the barcode combinations, around 1,500 cells with a median range of 2,500 and 11% collision rate can be read (20). The downstream analysis may result in cell clustering that distinguishes between distinct cell types in a mixed cell population and find peaks that are more or less accessible to particular cell types (21), thus identifying complicated cell populations, connecting regulatory elements to their target genes, and mapping regulatory dynamics during complex cellular differentiation processes through the chromatin regulatory landscape on the cell clusters (13).

The ability of macrophages to phagocytose and promote parasite growth makes them prime resident cells for *Leishmania*, although these cells do function as the primary effector cells in the clearance of infection (22). Epigenetic modifications and chromatin remodeling framework of macrophages with respect to *L. major* infection that causes CL are still poorly understood. Macrophage being the most prime target of infection may dictate disease fate, and the modular characteristics of parasite may influence the host macrophage plasticity for survival (23). A fine balance exists between different types of macrophages such as pro-inflammatory cytokine-expressing macrophages which mediate Th1-type response and anti-inflammatory cytokine-expressing macrophages having different subsets such as M2a, M2b, M2c, and M2d that elevate Th2-type response which regulates the parasite clearance (24). As a component and regulator of adaptive immunity, macrophages facilitate expression of interferon gamma (IFN-γ) and tumor necrosis factor-alpha (TNF-α), IL-12 that facilitates production of nitric oxide. To endure the hostile macrophage environment, *Leishmania* subverts the macrophage cellular process and metabolic pathway molecular functions and promotes chromatin remodeling to produce immunosuppressive molecules such as transforming growth factor β and IL-10 for its survival (25). One of the key mechanisms of parasite survival which we had reported was through governing IL-10 and IL-12 reciprocity. Through computational and systems biology integrated framework models, we had previously reported that epigenetic factors such as NFAT5 and their regulators, mainly SHP-1, may play deterministic roles in regulating IL-10 and IL-12 reciprocity (26).

In this study, we elucidate the epigenetic framework and chromatin remodeling dynamics using single-cell ATAC sequencing of *L. major*-infected RAW264.7 mouse macrophage cell lines at various time points. We have identified regulatory motifs governing the reciprocal control of cytokines, the parasite’s survival response, and infectivity. Our hypothesis posits that the observed shifts in population dynamics, cluster heterogeneity, and motif peak alterations are attributable to changes in the dynamic nature of chromatin accessibility. Additionally, we present, for the first time, quantified data at the single-cell level for macrophage-infected cells. *L. major* exhibits unique epigenetic modifications that are not reproduced by any other *Leishmania* spp. (27). Our previous findings had reported reciprocal expression of two crucial cytokines, IL-10 and IL-12, and we identified the time points of their distinct expression (26, 28). We introduce the term “sleepy macrophages” to characterize a subpopulation that does not express housekeeping genes but exclusively expresses novel gene and transcription factors. We also propose that precision-targeted therapeutics could modulate phenotype populations and potentially contribute to disease resolution.

## MATERIALS AND METHODS

### Sample preparation

Four samples were submitted for sc-ATAC sequencing. Mouse derived stable macrophage cell line RAW264.7 cells (1 × 10^6^) were infected with stationary-phase *L. major* promastigotes in 1:10 ratio and were incubated for 6, 12, and 18 h. Post-incubation, cells were washed thrice with phosphate-buffered saline (PBS) to remove unattached promastigotes, and cells were scrapped off and cryopreserved in cryomix [90% fetal bovine serum (FBS) + 10% dimethyl sulfoxide]; the control used for the study was uninfected RAW264.7 cells. To ensure maximum revival capacity of cells, they were frozen with gradual temperature changes: 0°C for 30 min, −20°C for 3 h, −80°C overnight, and liquid nitrogen storage for 24 h. Each sample was submitted to Neuberg Centre for Genomic Medicine, Supratech Reference Laboratory, in duplicate for nuclei isolation, microfluidics-based library preparation, and sequencing.

### Sample quality check

The cryovials were revived by thawing them at 37°C in water bath for 2 min, followed by mixing with 10-mL pre-warmed media and centrifuged at 300 rcf for 5 min. The cell pellet was resuspended in 1× PBS + 0.04% bovine serum albumin (BSA) (Sigma #A2153) and passed through 40-µm Flowmi Cell Strainer (Sigma NB.01). To check the viability of cells post-revival, 20 µL of the sample and 20 µL of 0.4% trypan blue (ThermoFisher #15250061) were gently mixed; 10 µL of the mix was loaded into Countess cell counting chamber slides (Thermofisher #A51876). Cells were counted using Countess 3 FL Automated Cell Counter (ThermoFisher).

### Nuclei isolation and quality check

Utilizing the CG000169 procedure from 10X Genomics, nuclei isolation was performed. The revived cells were centrifuged at 300 rcf for 5 min at 4°C. 100 μL lysis buffer containing 10 mM Tris-HCl (pH 7.4), 10 mM NaCl, 3 mM MgCl₂, 0.1% Tween 20, 0.1% Nonidet P40 substitute, 0.01% digitonin and 1% BSA was added and gently mixed 10 times, and the reaction mixture was incubated on ice for 5 min. To the lysed cells, 1-mL wash buffer [10 mM Tris-HCl (pH 7.4), 10 mM NaCl, 3 mM MgCl_2_, 1% BSA, and 0.1% Tween-20] was added and mixed gently five times followed by centrifugation at 500 rcf for 5 min at 4°C. The supernatant was discarded and nuclei pellet was resuspended in 7 µL of chilled nuclei buffer. The fifth portion of 10-µL nuclei suspension buffer was made with nuclei suspension and nuclei buffer and was mixed with 10 µL of 0.4% trypan blue stain, of which 10 µL of the mix was loaded onto the slide chamber. Nuclei concentration and viability were quantified using Countess 3 FL Automated Cell Counter.

### Transposition of isolated nuclei

Nuclei suspensions were incubated with transposase that was present in the transposition mix. Transposase fragments the DNA at open chromatin regions by entering the nuclei. Adapter sequences are simultaneously linked to the ends of the DNA fragments through polymerase chain reaction (PCR). The reaction mix was incubated at 50°C for 30 min, 37°C for 30 min, and 4°C for hold.

### Gel beads in emulsion and barcoding

Initiation of the steps was done by using CG000496 protocol. Briefly, barcoded gel beads, transposed nuclei, a master mix, and portioning oil loaded on a chromium Next GEM Chip H were combined to achieve single-cell resolution using Chromium iX. The distribution of the nuclei occurred at a limiting dilution, such that most (90%–99%) of the resulting gel beads in emulsion (GEMs) had one or no nuclei, while the majority of the rest did. The gel beads dissolved following GEM synthesis. After mixing and releasing from (i) an Illumina P5 sequence, (ii) a 16-nucleotide 10× barcode, and (iii) a Read 1 (Read 1N), oligonucleotides were further taken for thermal cycling to generate 10× barcoded, single-stranded DNA. The samples for thermal cycler-based extension were incubated at 72°C for 5 min, 98°C for 30 s, 98°C for 10 s, 59°C for 30 s, and 72°C for 1 min; this cycle took place with 12 times repetition and finally was kept at 15°C to hold.

### Single-cell library preparation and sequencing

During library preparation, P7 and a sample index wewere added through PCR. The P5 and P7 sequences used in Illumina bridge amplification were present in the final libraries. Following the manufacturer’s instructions, final libraries were quantified using a Qubit v.4.0 fluorometer (ThermoFisher #Q33238) and a DNA HS test kit (ThermoFisher #Q32851). We scanned the library on the Tapestation 4150 (Agilent) using high-sensitive D1000 screentapes to determine the insert size. Final QC checked libraries were sequenced on Illumina (Novaseq 6000) using S Prime (SP) flowcell at 50:8:16:50 cycles. Post-sequencing, the data were demultiplexed using the cell ranger arc v.7.0, and BCL2FASTQs were futher processed to generate RAW FASTQ, websummary files and clope file using mm10 reference genome.

### Clustering

Using log2 value as a filter parameter, custom filters were made to identify the population heterogeneity. We used Loupe browser for cell clustering. Peak calling is frequently repeated for each cluster in order to determine the accessible chromatin regions for various cellular populations. These regions are then the subject of a statistical test for correlations with different pre-defined genetic features. The primary objectives of downstream analysis techniques are to identify novel regulatory components and comprehend how they function within a cell (20).

### Dimensionality reduction for identification of principal components

A key technique for examining scATAC-seq data sets is through principal component analysis (PCA). Reduction of data dimensions helps in big data processing, interpretation, and identifying the uniqueness of each component. We used Factoextra and FactoMineR to perform PCA. We considered the data table as X and transformed it to the original coordinate system by orthogonal linear transformation. Let Fs (or Gs) stand for the vector representing the coordinates for the rows (or columns) on the axis of rank *s*. According to the transition formulas, these two vectors are connected and represented as


$$Fs\left(i\right)=1÷\sqrt{\lambda s}\;\;\sum_{k}xikmkGs\left(k\right)$$

$$Gs\left(k\right)=1÷\sqrt{\lambda s}\;\;\sum_{k}xikpiFs\left(i\right)$$

where Fs(*i*) is the coordinate of individual *i* on axis *s*; Gs(*k*) is the coordinate of variable *k* on axis *s*; λs is the eigenvalue associated with axis *s*; mk is the weight assigned to variable *k*; pi is the weight assigned to individual *i*; and xik is the catchall concept of the data table (row *i*, column *k*) (29). As the 6-h infected sample showed presence of unique clusters, we identified the principal components (PCs) from the clusters of 6-h sample.

### Gene set enrichment analysis

Gene set enrichment analysis (GSEA) enables evaluation of gene expression data at levels of gene sets, resolving the concerns arising due to insignificant statistics of gene expression post-analysis, non-unique biological activity, identifying single gene function in cellular process, and preventing overlaps among phenotypes (30). Identification of gene sets to be involved with a specific biological activity, gene ontology, molecular function, or pathway, is compared to the ranked gene list. Determination of the enrichment score (ES), leading edge subset of genes, and gene rank was performed (31). We evaluated 814 genes from the 6-h sample in order to provide physiologically pertinent details regarding the differential expression of genes belonging to sleepy macrophages. The clusters were classified into two groups: sleepy macrophage (Clusters 3 and 6) and normal (Clusters 1, 2, 4, and 5) (Class 1 and 0, respectively), which were investigated for differential expression and enrichment.

### Differential correlation analysis

It is critical to understand how the correlation between molecules under two different experimental conditions has changed, in addition to how the mean amounts of molecules in the omics data have changed. We employed DiffCorr package to understand the correlation among the clusters in the samples. For each data set, DiffCorr generates correlation matrices, locates the first principal component-based “eigen-molecules” in the correlation networks, and uses Fisher’s *z*-test to assess differential correlations between the two groups (32). We performed differential correlation analysis for the 6-h sample.

### Construction of transcription factor-target gene network

As the transcription factors which are expressed in Cluster 3 may result in upregulating the genes from the same cluster, we prepared an integrated framework network of the gene targets and transcription factors enriched in Cluster 3. We used the TFlink database, which offers thorough and extremely reliable information on transcription factor-target gene (TFTG) interactions for *Mus musculus*. It integrates information from other TF databases, including JASPAR and TRRUST database, to offer cumulative statistics for the TFs for a specific organism.

The entire integrated biomolecular interaction network of TFTG was constructed and analyzed using Cytoscape (v.3.6.0). The initial TFTG network had 814 genes obtained from Cluster 3 and 21 transcription factors. The constructed TFTG network consisted of 476 nodes and 1,060 edges. This initial network was then subjected to simulated annealing algorithm in Cytoscape, which will give a robust inter-regulatory TFTG network that is resilient and comprehensible in nature as the loosely connected edges of the network are filtered out. The most clustered or heavily weighted nodes are positioned at the bottom of the network using the simulated annealing process, which analyzes each node in the network. In contrast, nodes with lesser clusters are arranged in descending order in the upper part of the network.

The robust simulated annealing network obtained is further analyzed considering the potential of Cytoscape plugin: CytoHubba. CytoHubba uses a double screening scheme for ranking nodes and edges in a network. Further network analysis through CytoHubba helps us understand the function of an individual node and its collaboration with other nodes in a cluster. It uses algorithms like betweenness centrality, closeness centrality, degree of nodes, maximal clique centrality (MCC), clustering coefficient, bottleneck, and others to present a condensed and more robust nature of the inter-regulatory transcription factor network with their putative target genes.

### Cell cycle analysis and effect on cell-cell communication

RAW264.7 cells were infected with stationary-phase *L.major* promastigotes for 6 h. They were washed thrice with PBS and fixed with 70% ethanol for 30 min at 4°C. Cells were later washed thrice with PBS to which RNase A (100 µg/mL, 0.1% Triton X and propidium iodide 50 µg/mL) was added and incubated for 15 min at room temperature (RT). Cells were acquired on BD New Canto II FACS analyzer.

By analysis of *k*-means of 6-h infection sample, we identified HOXA9 as the most statistically significant protein having the highest expression with a log2 fold change of 4.74. We identified the genes that are regulated by this protein and identified 7,241 genes. We mapped the ontologies associated with these genes using Consensus PathDB. Furthermore, we analyzed the expression of H2-D1 as it is the MHC-II associated with RAW264.7 cells. The log2 fold and motif accessibility were also investigated only to reveal the association of MHC-II with the cell cycle of the host.

### Western blot analysis of peritoneal macrophages

Peritoneal macrophages were isolated from the peritoneal cavities of female (3–4 weeks old) Balb/c mice. Cells were adhered for 24 h in DMEM supplemented with 10% FBS and washed with PBS to remove non-adherent cells. Stationary-phase *L. major* promastigotes were used in 10:1 ratio to infect macrophages at different time points (1, 6, 12, 18, and 24 h). Further cells were scraped and washed with PBS thrice. Radioimmunoprecipitation assay buffer (RIPA) buffer was used to isolate protein from these samples. Protein estimation was done using bicinchoninic acid (BCA) assay. A total 20 µg of whole protein was loaded on 15% gel, which was further transferred on nitrocellulose membrane. The membrane was blocked with 3% BSA for an hour, which was later washed thrice with TBST. IL-10 [ThermoFisher Scientific (PA595561)] (1:1,000) and IL-12p40 [ThermoFisher Scientific (PA579461)] (1:1,000) primary antibodies were used for analysis. The blots were incubated for 18 h at 4°C. Following incubation, the blot was washed three times with TBST before being exposed to the secondary antibody, anti-rabbit IgG (whole molecule)-peroxidase antibody made in goat [Sigma-Aldrich (A9169)], which was incubated at room temperature for 1 h. Luminol-Enhancer Solution [Cyanagen (XLS070L)] and H_2_O_2_ [Cyanagen (XLS070P)] were used to develop the blot, and Amersham ImageQuant 800 imager was used to capture the images. Image J was used for densitometric analysis.

### Confocal analysis of SHP-1 and NFAT5 at 6 h of infection

RAW264.7 cells was seeded on eight-well chamber slides. These were infected with stationary-phase *L. major* promastigotes for 6 h. Later, cells were washed with 1× PBST (PBS in 0.1% Triton X) and fixed with 4% paraformaldehyde for 20 min at RT. After washing the cells thrice with 1× PBST, the cells were permeabilized with 1× PBST for 10 min and blocked with 3% BSA for 30 min. SHP-1 [Cell Signaling Technology (3759S)] and NFAT5 [ThermoFisher Scientific (PA1-023)] primary antibodies were added to the cells and were incubated at RT for 2 h (1:1,000). Cells were washed thrice with PBST and later incubated with anti-rabbit IgG (H + L) F(ab′)2 fragment (AlexaFluor 488 Conjugate) [Cell Signaling Tachnology (4412S)] (1:500) and Mouse Phalloidin AlexaFluor 568 [ThermoFisher Scientific (A12380)] (1:500) for 1 h. Post-incubation, cells were washed with 1× PBST thrice and counterstained with 4',6-diamidino-2-phenylindole (DAPI) for 10 min. Cells were later washed with 1× PBST thrice and with distilled water thrice. After air-drying the cells, mounting media [ThermoFisher Scientific (00-4958-02)] was used to mount the slides and were visualized at 60× and 100× on Zeiss LSM 880 with Airyscan microscope. Fiji was used for analysis of images.

## RESULTS

### Single-cell ATAC sequencing reveals diverse macrophage clusters post*-L. major* infection

A total of 2,262 cells were captured for uninfected cells, 1,886 cells from the 6-h infected sample, 10,835 cells from the 12-h infected sample, and 6,411 cells from the 18-h infected sample. Based on the log10 fragment value by depth, cells were clustered. Median high-quality fragments per cell for uninfected cells were 7,722; those for the 6-h infected cells were 32,590; those for the 12-h infected cells were 9,447; and those for the 18-h infected sample was 11,792 (Fig. S1).

After analysis of all the samples, 6 cell clusters were observed in the control and the 6-h sample, 12 clusters in the 12-h sample, and 10 clusters in the 18-h samples (Fig. S1 and S2). These clusters exhibited varying cell counts with diverse percentage distribution of macrophage population. There was an increase in the number of cell clusters from 6 to 12 h, although the number of cluster decreased at 18 h as compared to the 12-h sample. Also, the profiles of control and samples infected with *L. major* showed different clustering patterns, which differentiate infected and non-infected characteristics (Fig. S1 and S2).

### Percentage population of pro-inflammatory cytokine and anti-inflammatory cytokine-expressing macrophages changes with time point of infection

**Fig 1:**
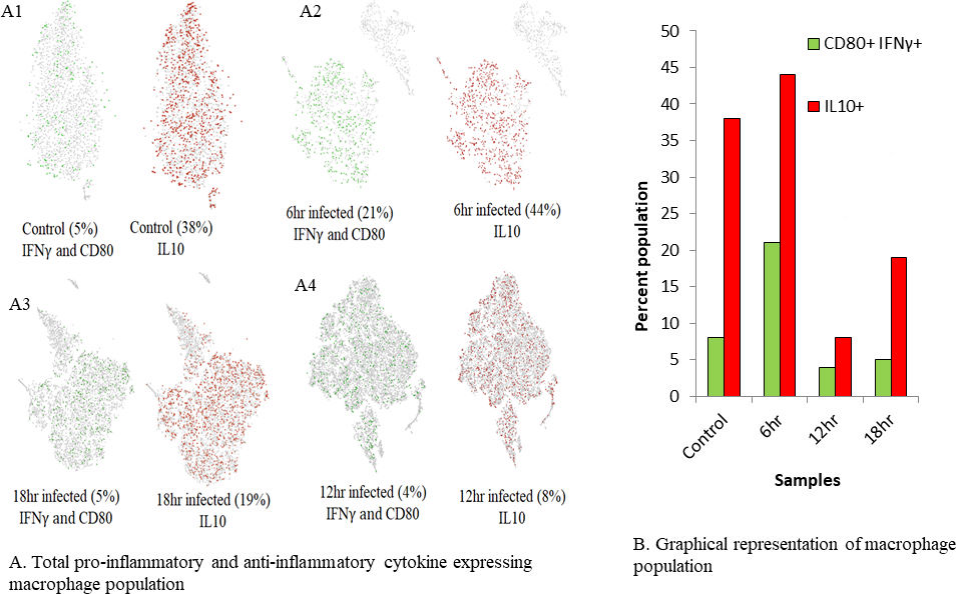
(A) Expression of parasite eliminating phenotype and parasite survival promoting phenotype markers IFN-γ, CD80, and IL-10 in samples (CD80 IFN-γ -expressing cells are represented in green color and IL-10 -expressing cells in red). (A1) Control, (A2) 6 hr, (A3) 12 hr, and (A4) 18 h. (B) Graphical representation of macrophage types at different time points of infection.

Subtypes macrophages were identified based on their differential expression levels for which threshold for marker genes were set with cutoff log2 of >0.5. For identification of pro-inflammatory cytokine-expressing macrophages, we used IFN-γ and CD80 as markers as they may characterize parasite-eliminating response (24). Anti-inflammatory cytokine-producing macrophage subtypes commonly express IL-10; hence, to get an overview of parasite survival promoting response, we used IL-10 as a marker (24) for which threshold was set with a cutoff log2 of >0.5 (Fig. 1A1 through A4). Parasite-eliminating subsets are generally M2 subtypes, which include M2a, M2b, M2c, and M2d. These were characterized by expression of cell markers. Parameters for M2a were set as log2 CCl24 of >1 and log2 SOCS3 of >1; for M2b, log2 TNF of >1 and log2 CCl1 of >1; for M2c, log2 TGF-β of >1 and log2CD163 of >1; and for M2d, log2 NOS2 of>1 and log2 CCl5 of >1 (28) (Fig. S3). We could see the shift in macrophage population with time points of infection where IL-10-expressing macrophages and M2a population were most dominant in all the samples; IFN-γ- and CD80-expressing population was most dominant in the 6-h infected sample; and M2d was enriched in the 6-h infected sample as well. In the 12-h infection sample, a decline in the number of cells was observed for all the macrophage types, which later at 18 h was followed by an increase in IL-10-expressing macrophage subtype (Fig. 1B; Fig. S3).

### Infection of macrophages with *L. major* differentiates IL-10 and IL-12 production population

**Fig 2:**
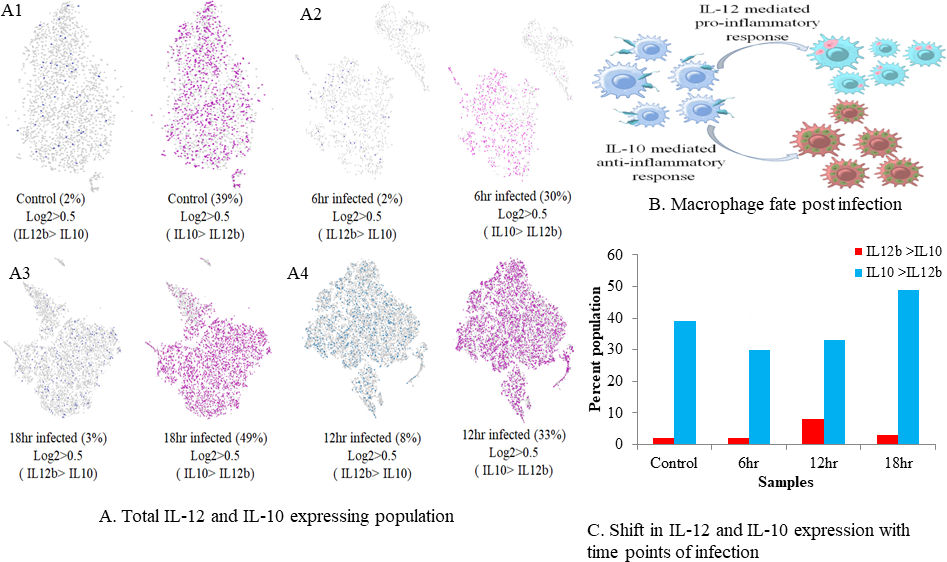
Identification of the reciprocal relationship between IL-10 and IL-12. (**A**) Macrophage-expressing IL-12 more than IL-10 and vice versa in all the samples. (A1) Control, (A2) 6 h, (A3) 12 h, and (A4) 18 h. (**B**) Abstract highlighting the fate of macrophages post-infection with *L. major* if IL-12 and IL-10 are secreted. (**C**) Change in expression patterns of IL-10 and IL-12 with time.

Macrophages may express IL-12, a pro-inflammatory cytokine, and IL-10, an anti-inflammatory cytokine, in response to *L. major* infection (Fig. 2B). Populations were identified based on unique expression of IL-12 or IL-10. This categorization identified the cellular response to infection with time. The parameter used was log2 IL-12b of >0.5 and log2 IL-10 of <0.5 for distinguishing the IL-12-producing group from the IL-10-producing group for which parameter for filtering was set as log2 IL-10 of >0.5 and log2 IL-12b of < 0.5 (Fig. 2A1 through A4). In the uninfected sample, we observed that populations expressing IL-10 were more than IL-12-expressing cells, although we observed slight changes in depletion of IL-10-producing cells at 6 h of infection. At 12 h, IL-12-expressing cells increased but did not dominate over IL-10-expressing cells, and they diminished at 18 h (Fig. 2C). We could deduce that perhaps, at 6 and 12 h post-infection, macrophages attempt to inhibit IL-10 production in favor of promoting IL-12 production. However, due to modulatory effects of intracellular *L. major* on regulation of host cytokine machinery, macrophages may fail to express IL-12 over IL-10, resulting in a subsequent decrease.

### Reciprocal regulation of IL-10 and IL-12 through NFAT5 and SHP-1 expression

**Fig 3:**
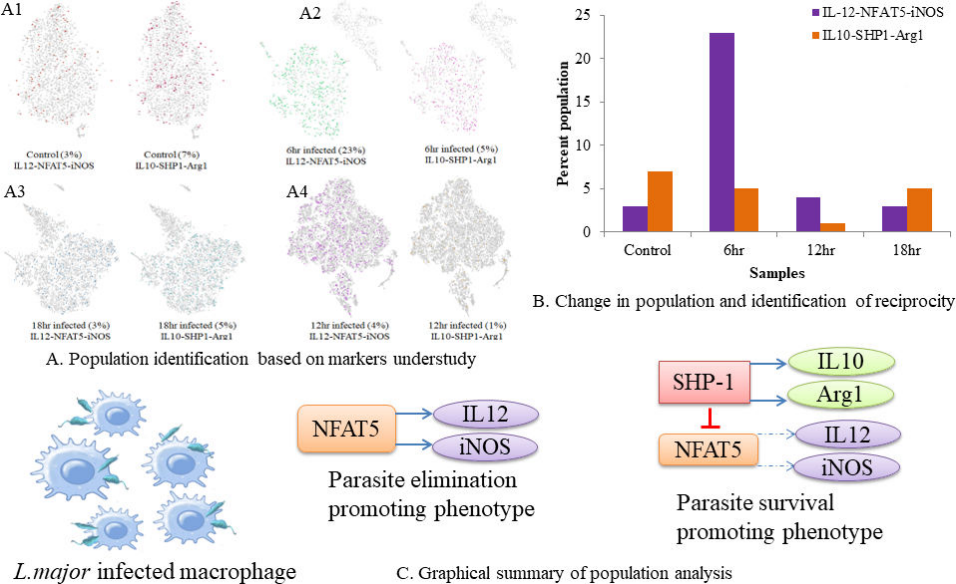
Identification of reciprocity in IL-10 and IL-12 expression patterns through NFAT5 and SHP-1. (**A**) Populations expressing IL-12, NFAT5, and iNOS versus populations expressing IL-10, SHP-1, and arginase1. (A1) Control, (A2) 6 h, (A3) 12 h, and (A4) 18 h. (**B**) Graphical representation highlighting the reciprocal relationship between IL-10 and IL-12 mediated by NFAT5 and SHP-1. (**C**) Graphical abstract of the aim behind the analysis.

From our previous findings, we had already reported that upon *L. major* infection, NFAT5 might govern expression of IL-12 and nitric oxide. Furthermore, SHP-1 may regulate NFAT5 by dephosphorylating it at the auxiliary export domain. This inhibits the NFAT5-dependent pro-inflammatory response and steers the cellular machinery toward IL-10 and arginase-mediated ornithine cycle. (Fig. 3C). We identified if these deterministic genes are expressed together to get an insight into the IL-10 and IL-12 expression axis. Parameters used to identify these populations across samples were set as log2 NFAT5 motif of >0.5, log2 IL-12b sum of >0.5, and log2 NOS of >0.5. For the second group, the distinguishing parameters were set as log2 IL-10o of >0.5, log2 ptpn6 of >0.5, and log2 Arg1 of >0.5 (Fig. 3A1 through A4). We could discern that at 6 h of infection, the parasite-eliminating population was dominating over parasite survival-favoring population. At 12 h, population architecture dropped without changing the dynamics; however, at 18 h, the dynamics changed with subtle shift of parasite survival-favouring population over parasite-eliminating population (Fig. 3B).

### Infection with *L. major* for 6 h identifies sleepy macrophages

**Fig 4:**
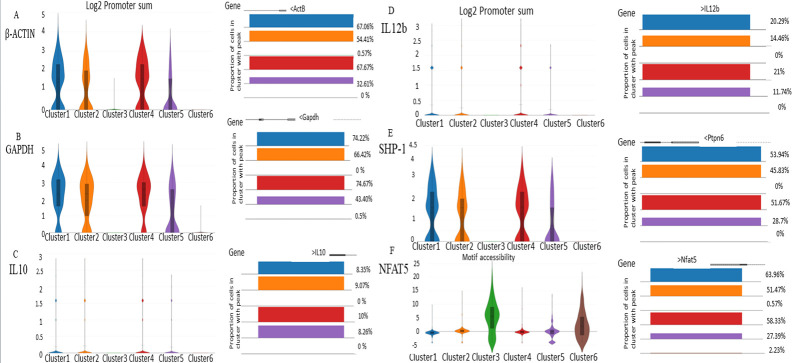
Differential expression of genes in 6-h sample in all clusters. (**A**) Violin plot of feature expression and peaks of ActB. (**B**) Violin plot of feature expression and peaks of GAPDH. (**C**) Violin plot of feature expression and peaks of IL-10 (**D**) Violin plot of feature expression and peaks of IL-12b. (**E**) Violin plot of feature expression and peaks of SHP-1. (**F**) Violin plot of NFAT5 motif accessibility and peak of NFAT5.

As we observed the changes in gene expression pattern which shifted the cell population dynamics from parasite-eliminating group to parasite survival cell subset starting at 6 h post-infection, we analyzed the chromatin accessibility of IL-10, IL-12p40, SHP-1, and NFAT5 and used B-actin and GAPDH as housekeeping gene control. To our surprise, there were two clusters (Clusters 3 and 6) which showed minimal expression of housekeeping genes and genes under study (Fig. 4A through E) (Table 1). This made us intrigued as no other clusters from other samples showed a similar expression pattern. These cells are alive; the chance that these undergo a survival mechanism in order to combat the infection was fascinating. As these macrophages were not expressing housekeeping genes, genes which we were studying, and any marker genes which macrophages normally express, we named them as sleepy macrophages to describe their dormancy. NFAT5 motif, which regulates IL-12 and IL-10 expressions, was highly enriched in sleepy macrophages (Fig. 4F), indicating potential involvement through molecular mechanisms, cellular processes, and biological signaling in sleepy macrophages to regulate the reciprocity of IL-10 and IL-12. Results for all samples are shown in Fig. S4.

**TABLE 1:** Expression and accessibility of gene promoters under study

Gene	Median expression Cluster 3	Mean expression Cluster 3	Maximum expression Cluster 3	Percent peak range Cluster 3	Median expression Cluster 6	Mean expression Cluster 6	Maximum expression Cluster 6	Percent peak range Cluster 6
B-actin promoter sum	0	0.0090	1.58	0.571429	0	0	0	0
GAPDH promoter sum	0	0	0	0	0	0.00885	1.58	0.558659
IL-10 promoter sum	0	0	0	0	0	0	0	0
IL-12 promoter sum	0	0	0	0	0	0	0	0
SHP-1 promoter sum	0	0	0	0	0	0	0	0
NFAT5 promoter sum	0	0.00905	1.584	0.571429	0	0.0354	1.584	2.234637
NFAT5 motif	5.682141	5.28775	22.22298	N/A	1.745482	2.316064	18.40887	N/A

### Identification of principal components from sleepy macrophages

**Fig 5:**
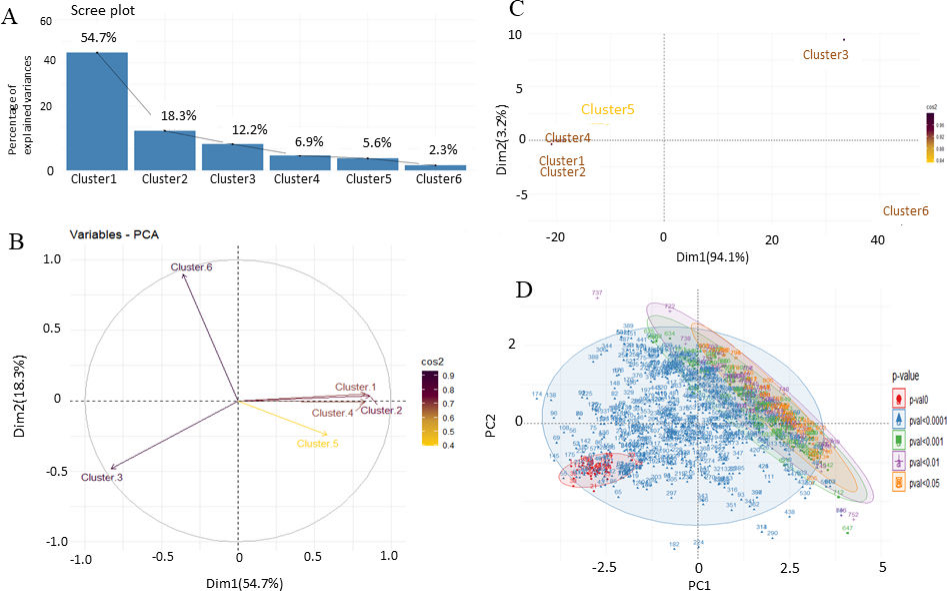
Principal component analysis of 6-h sample by differential expression of all the clusters. (**A**) Scree plot, (**B**) variables in clusters, (**C**) PCA of clusters, and (**D**) PCA based on *P* value.

To discern the enriched genes in Clusters 3 and 6, we extracted the entire feature expression set from the 6-h sample. It was noted that the genes exhibiting upregulation in Clusters 3 and 6 are markedly downregulated in Clusters 1, 2, 4, and 5, as detailed in Table S1. The scree plot analysis disclosed an elbow point at Cluster 3, beyond which the slope became relatively flat. This observation indicates that variables from Clusters 4–6 exhibited low variance. Notably, Clusters 1–3 collectively account for 85.2% of crucial variables, suggesting their suitability for further investigation to identify principal components, as illustrated in Fig. 5A. As a set of variables, Cluster 3 is observed in the negative quadrant, indicating that features from Cluster 3 may exhibit a negative correlation with variables from other clusters. (Fig. 5B). When individual genes were analyzed as eigenvectors, Cluster 3 displayed higher cos2 values in the positive quadrant (Fig. 5C). The significance of individual features as principal components is determined by the magnitude of their cos2 values; a higher cos2 value signifies greater importance. Subsequently, we identified principal components based on their level of significance from all the clusters (Table S3). PCs within the ellipses are notably contributing to the infection at 6 h, given the elevated number of highly significant principal components, as depicted in Fig. 5D, we proceeded to categorize the clusters for gene set enrichment. This approach aimed to identify potential components within crucial clusters that may play a regulatory role in the infection post-6 h of infection.

### Gene set enrichment and differential correlation of sleepy macrophages

**Fig 6:**
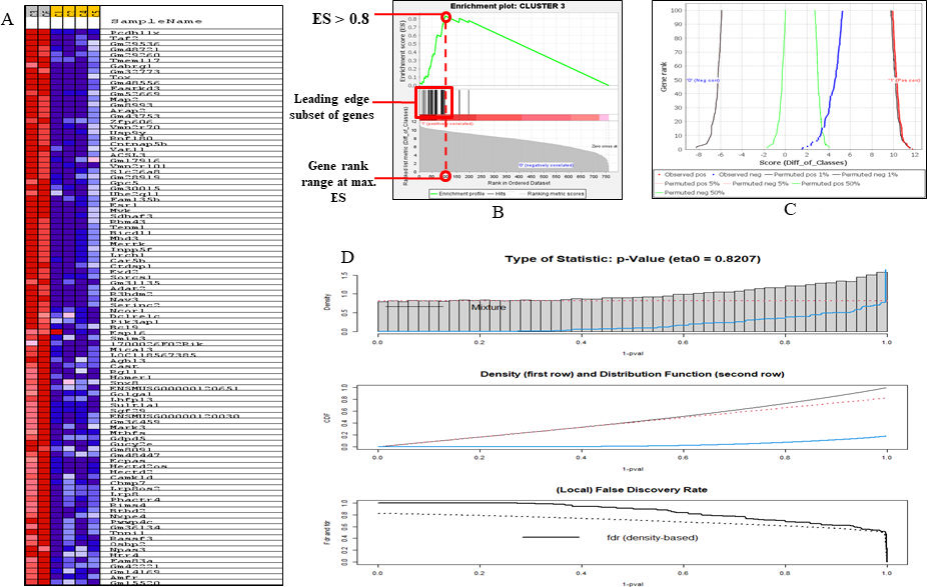
Gene enrichment analysis from Clusters 3 and 6 of 6-h sample. (**A**) Heat map of genes enriched from Clusters 3 and 6. The heat map visually displays the genes within the leading edge subsets after clustering. In this representation, gene expression values are depicted using a color spectrum where the variation in colors (ranging from red to pink, light blue to dark blue) corresponds to the diversity in expression levels (high, moderate, low, and lowest). (**B**) Enrichment plot of Cluster 3. (**C**) Butterfly plot of Cluster 3. (**D**) Differential correlation of Clusters 3 and 6 with Clusters 1, 2, 4, and 5.

A heat map is employed to illustrate the expression of clustered genes within leading-edge subgroups. The correlation between ranked genes and the assigned class is visually represented on a heat map for each phenotype. In this instance, the heat map reveals that Clusters 3 and 6 exhibit elevated expression of the leading-edge subset of genes, indicating a correlation with the sleepy macrophage phenotype when compared to other clusters in the data set (Fig. 6A). In the enrichment plot, the magnitude of the increment in the enrichment plot depends on how well the gene correlates with the defined phenotype; the graph illustrates the positive enrichment of gene sets from Cluster 3 with an ES of greater than 0.8. Additionally, it was observed that the leading-edge subset of a gene set contributed to the sleepy macrophage phenotype as they were correspondingly located in the area under the ES plot. Furthermore, the bottom-most portion of the enrichment plot shows the value of the ranking metric which measures every gene’s correlation in the gene set with the defined phenotype. The ranking metrics’ value goes from positive to negative as we move down the ranked list, with a positive value indicating correlation with the first phenotype sleepy macrophage and a negative value indicating correlation with the second phenotype (normal). With respect to the ES, the highest-ranking genes from the gene set fall between ranks 0 and 100 (Fig. 6B).

The butterfly plot provides a means to visually represent how permutations of the data set impact the correlation between gene ranking and the score assigned by the ranking metric. The observed correlation for the top genes, as well as permuted (1%, 5%, and 50%) positive and negative correlations, was calculated for the data set. The plot illustrates that 100 genes associated with the normal phenotype exhibit a negative correlation to sleepy macrophage, and the top-ranked genes shift more toward the sleepy macrophage phenotype, thereby providing support for the enrichment plots (Fig. 6C). Moreover, to observe the differential correlation pattern change in gene expression between Clusters 1, 2, 4, and 5 and Clusters 3 and 6, we performed (Table S7) differential correlation analysis using Pearson’s correlation coefficient and observed density (*P* value) and distribution function to be correlated, with false discovery rate to be decreasing with *P* value (Fig. 6D).

### TFTG network links cellular pathways and novel markers in sleepy macrophages

Our comprehensive study identified 51 transcription factors enriched in sleepy macrophages which may regulate 814 identified genes (Table S2); we could map 22 transcription factors and their target genes from sleepy macrophages genes and constructed the network, which had 476 nodes and 1,060 edges (see Fig. 8B and C; Table S6). The network was simulated in cytoscape as well as in simulated annealing algorithm environment. The network distribution was made lucid by reducing the number of multi-edge node pairs and weakly connected edges, making the network more robust (Table 2).

**TABLE 2:** Statistical analysis of transcription factor-target gene inter-regulatory network after simulated annealing representing a decrease in the multi-edge node pairs, making the network more robust by filtering out the loosely connected edges

Parameters	Original network values	Simulated annealing network values
Clustering coefficient	0.005	0.005
Connected components	1	1
Network diameter	8	8
Network radius	4	4
Shortest paths	226,100 (100%)	226,100 (100%)
Characteristic path lengths	3.556	3.556
Average number of neighbors	4.454	4.454
Number of nodes	476	476
Network density	0.009	0.009
Isolated nodes	0	0
Number of self-loops	0	0
Multi-edge node pairs	16	0

**Fig 7:**
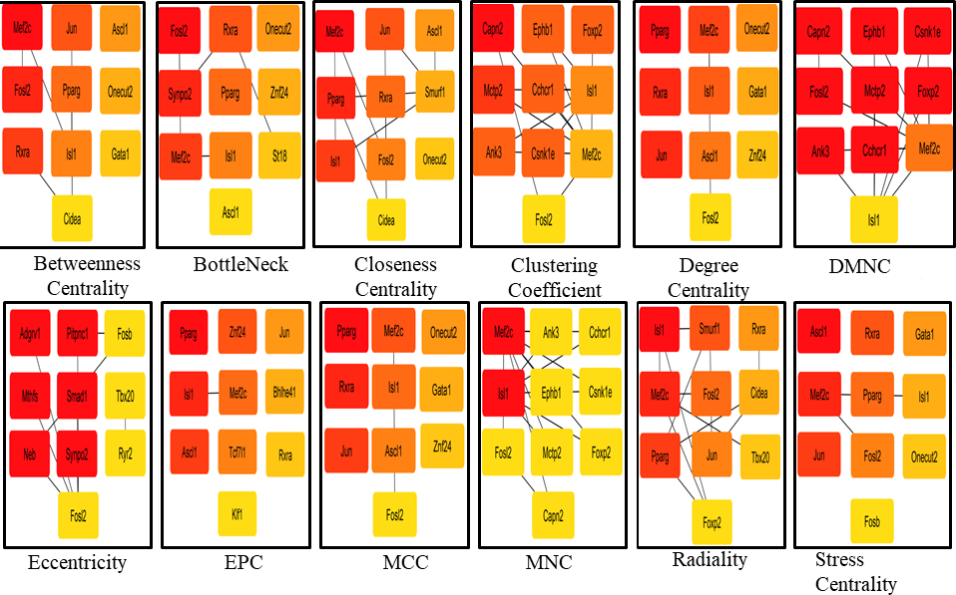
Top-ranking genes based on 12 scoring techniques were identified from the leading inter-regulatory TFTG network. Red signifies the highest score; orange signifies moderate score; and yellow signifies lower score.

**Fig 8:**
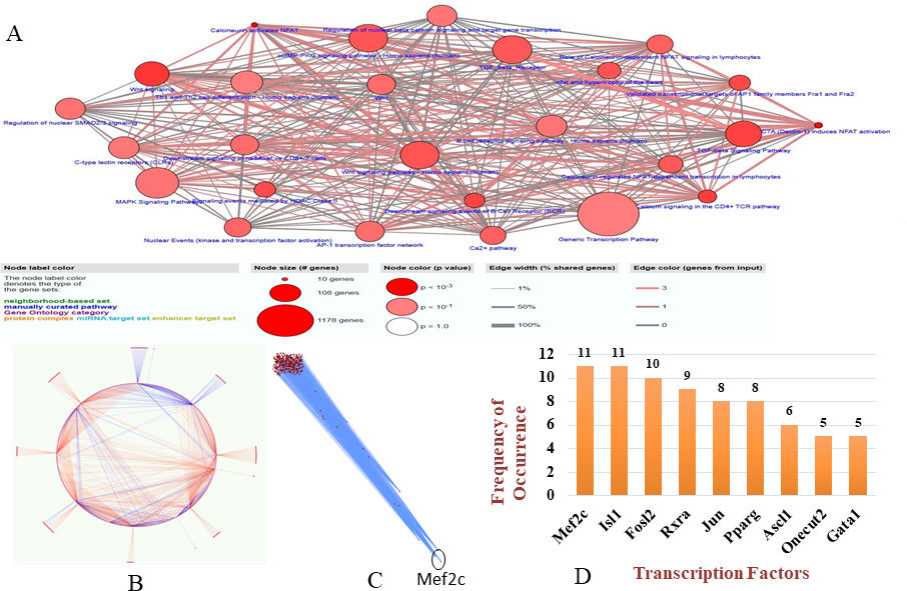
Transcription factor-target gene (TFTG) network analysis. (**A**) Pathway enrichment of sleepy macrophage genes and transcription factors (*P* value < 0.01). (**B**) The simulated network’s circular layout demonstrating the strength of chosen transcription factors over the whole network. (**C**) The inter-regulatory TFTG network after running the simulated annealing algorithm, showing placement of heavily weighted nodes (TFs) positioned at the bottom of the network. (**D**) The top five ranked transcription factors are represented graphically, based on their frequency of occurrence according to the 12 scoring techniques of CytoHubba plugin.

The significant transcription factors in the inter-regulatory TFTG network were identified using the Cytoscape plugin, CytoHubba. It was used to find the most essential modules and top 10 ranked nodes in the entire network. The 12 scoring methods used by CytoHubba to determine the critical network modules and top-ranked TFs/TGs in the inter-regulatory network include betweenness centrality, bottleneck, closeness centrality, clustering coefficient, degree centrality, eccentricity, edge percolating coefficient, MCC, density of maximum neighborhood component, maximum neighborhood component, radiality, and stress centrality (Fig. 7). The top 10 nodes in the network were determined by their frequency of occurrence in each scoring method, respectively. Mef2c, Isl1, Fosl2, Rxra, Jun, Pparg, Ascl1, Onecut2, and Gata1 were observed to be significantly enriched, out of which Mef2c and Isl1 were the two most critical transcription factors having the highest frequency of occurrence in the *L. major* infection (Fig. 8D). The ontologies associated with the top 10 transcription factors highlighted upregulation of NFAT5-mediated signaling, Th1 and Th2 cell differentiation, and histone deacetylase (HDAC)-mediated mitogen-activated protein kinase (MAPK) signaling (Fig. 8A).

### Cell cycle arrest mediated by TP53 regulation interferes with MHC-II expression in sleepy macrophages

**Fig 9:**
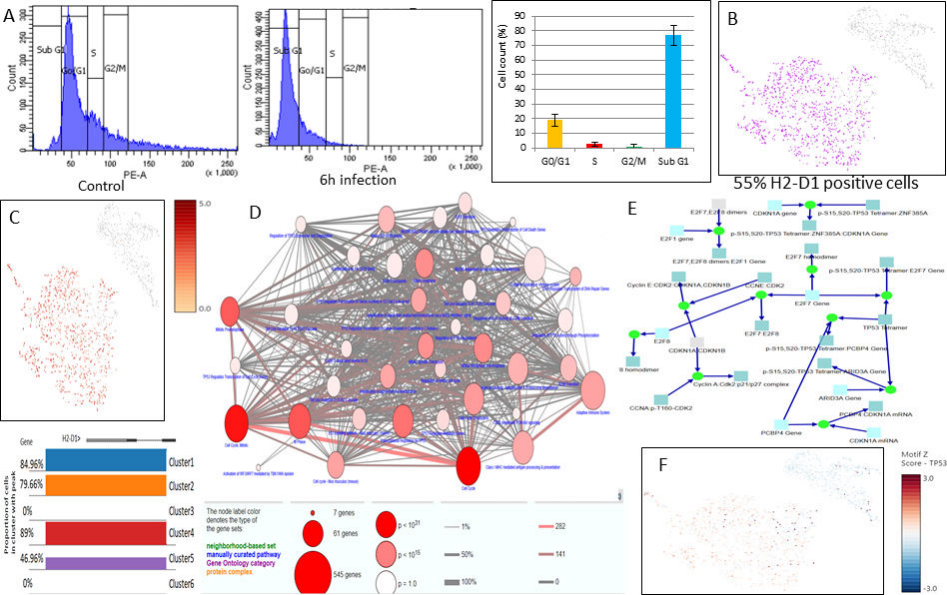
(**A**) Cell cycle analysis of RAW264.7 cells infected with *L. major* for 6 h and identification of percentage of population (results obtained are from three individual experiments). (**B**) t-distributed stochastic neighbor embedding (t-SNE) map of H2-D1 expression. (**C**) Promoter accessibility and motif *z*-score of H2-D1 population. (**D**) Gene ontology associated with HOXA9 (*P* value < 0.001). (**E**) Cell cycle analysis of sleepy macrophages shows reactions favoring G0/G1 phase. (**F**) TP53 motif accessibility and expression at 6 h post-infection with *L. major* (*P* value < 0.01).

Cell cycle analysis of 6h infected sample identified 18.86% of cells in the G0/G1 phase, 2.53% of cells in the S phase, 1.11% of cells in the G2/M phase, and 76.88% of cells in the sub-G1 phase (Fig. 9A). *k*-Means analysis identified 51 genes which regulate sleepy macrophages, and they were associated with gene transcription and sumoylation (data not shown). Additionally, we aimed to investigate whether sleepy macrophages could engage in cell-cell communication through Signal 1, which is MHC-II and TCR/BCR interactions. We observed that H2-D1 allotype, which is expressed in the RAW264.7 cell line, was downregulated in sleepy macrophages, suggesting a silent defense mechanism of parasites which may deform host antigen processing mechanisms in order to process pathogen-associated molecular pattern molecules (PAMPs) of pathogen to activate other immune cells toward Th1 response (Fig. 9B and C). Furthermore, to see the connections associated with regulation of MHC-II expression, we analyzed the genes according to *k*-means and identified that TP53 regulation-mediated G1 and G2 arrest is prevalent in sleepy macrophages (Fig. 9E). The expression of TP53 was irregular in sleepy macrophages (Fig. 9F). When we analyzed the genes correlated with HOXA9, which was most abundantly and significantly expressed in sleepy macrophages, we found that TLR-mediated MyD88 pathway, cell cycle genes, cell cycle-associated genes, and TP53 expression and regulation were highly associated with sleepy macrophages (Fig. 9D).

### Expression of NFAT5 and SHP-1 post-6-h infection with *L. major*

**Fig 10:**
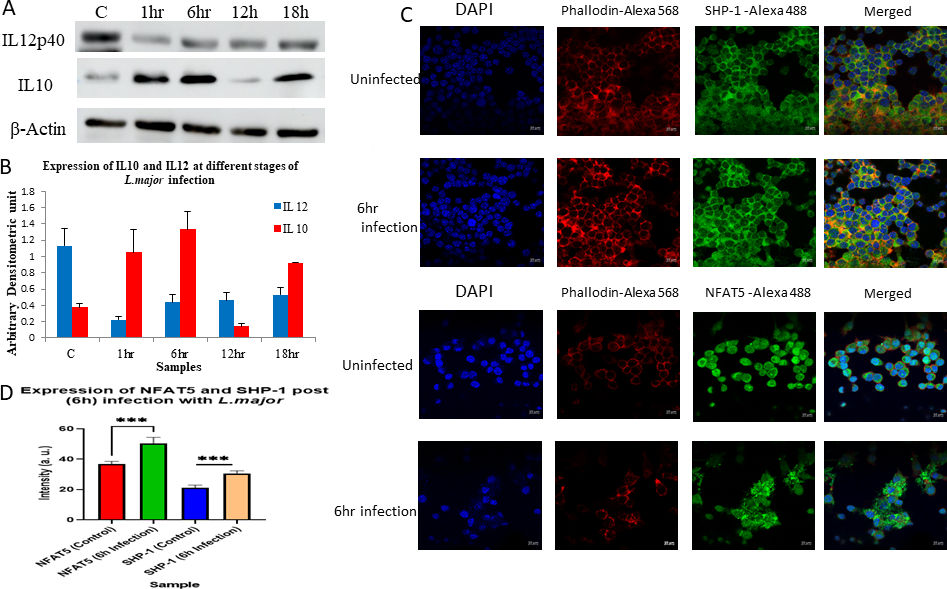
(**A**) Expression analysis of IL-10 and IL-12p40 on peritoneal macrophages infected with *L. major* at different time points. (**B**) Densitometry analysis of blots. (**C**) Localized expression of SHP-1 and NFAT5 in RAW264.7 cells. (**D**) Intensity measure of expression of SHP-1 and NFAT5 (*P* value < 0.01).

Expression of IL-10 and IL-12 was observed to have undergone alterations with time. Post-6 h, IL-12 levels had increased with even more higher expression of IL-10, and at 12 h, we observed that expression of both proteins had reduced followed by a shift in dynamicity where IL-10 expression had increased. Data from immunoblotting of peritoneal macrophages show similarity to the trend of expression in sc-ATAC time points, which was deciphered from RAW264.7 cells (Fig. 3A, B, 10A and B;Table S8). Localized expression of NFAT5 and SHP-1 at 6 h post-infection was significantly higher as compared to the uninfected sample, where NFAT5 expression at 6 h post-infection was highest as compared to other groups (Fig. 10C and D) and which also corroborates with (Fig. 4).

## DISCUSSION

### The dynamics of macrophage cell clustering reveal notable shifts, depending on the time point of infection

As compared to control samples, the 6-h sample showed a similar number of clusters, although the dynamics of clusters shifted in the t-SNE plot with changes in the number of cells in individual clusters. At 12 h, cluster size and number increased with cell distribution among the 12 clusters. At 18 h, cluster number changed to 10. We do not have any apparent reason for this change; we predict that the macrophage plasticity was modulated by the parasite so that number of cells in a particular cluster increases so the number of phenotypic macrophages decreases.

### Reciprocal relationship between IL-10 and IL-12 was observed during *L. major* infection

Two main phenotypes of macrophages are pro-inflammatory cytokine-expressing macrophages and anti-inflammatory cytokine-expressing macrophages, which offer inflammatory and regulatory roles, respectively, in leishmaniasis. They differ mainly in production of chemokines and cytokines, transcription factors, and specific markers, which identify their function (24). IFN-γ is expressed in pro-inflammatory cytokine-expressing macrophage phenotypes (33); CD80 is an expression marker of these macrophages (34), whereas IL-10 is commonly expressed in all anti-inflammatory cytokine-expressing macrophage subtypes (24, 35). Hence, we used them to identify overall parasite-eliminating and parasite survival-promoting macrophage subtypes at all time points and observed the dominance of anti-inflammatory cytokine-expressing macrophages over pro-inflammatory cytokine-expressing macrophage populations which might be attributed to amastigote-driven modulation of macrophage plasticity with the time of infection. Identification of both macrophage subsets was one of our novel findings as categorization of macrophage subtypes was possible through sophisticated parameters provided by scATAC-seq. Typically, log2 values harnessed are set as more than 0.5 (36), but we set threshold as 1 to ensure capturing of specific subtypes of macrophages and to avoid identification of mixed population. M2a enrichment suggested that IL-10 and arginase were overexpressed and inducted. Increased IL-10 levels and decreased IL-12 expression have been demonstrated by M2d enrichment.

### IL-10 expression appears to be more prevalent in *L. major* infection

From the macrophage subtype expression paradigm, we wanted to identify the populations which were potent in expressing IL-10 and IL-12. Hence, we filtered the population which either expressed IL-10 or IL-12 to avoid overlap of populations. The sudden increase of IL-12 producing macrophages at 12 h post-infection suggested that macrophages were trying to combat infection; nonetheless, their population subsided at 18 h due to overexpression of IL-10 by the majority of the population.

### Reciprocal modulation in the expression of IL-10 and IL-12 appears evident following 6 h of infection

Using systems-based discrete mathematical models, we had previously reported about the modulation of IL-10 and IL-12 by NFAT5 and SHP-1 in *L. major* infection models. In order to facilitate the elimination of parasites, NFAT5, a transcription factor and chromatin remodeling inducer, modifies nucleosomes 1 and 2 of the IL-12b gene and the IL-10 promoter region. Later, as a mechanism to combat infection and regulate pro-inflammatory cytokine, *Leishmania* may modulate host machinery to activate SHP-1, a phosphatase, and to inactivate NFAT5, and therefore the chromatin architecture changes entirely, which activates the IL-10 gene (26). Our systems-based findings were validated when we observed expressional changes in IL-10 and IL-12 expression post*-L. major* infection from 1 h post-infection until 48 h, with major changes occurring at 6, 12, and 18 h (28).

The expression-based findings from scATAC-seq data corroborate with our data. We checked co-expression of the parasite-eliminating group, which included IL-12, NFAT5, and iNOS genes, comparatively with the parasite survival-favoring group, which included IL-10, SHP-1, and Arg1. Since nitric oxide production ultimately determines the fate of parasite survival, expression of iNOS and arginase was also taken into consideration while clustering. At 6 h, we observed parasite-eliminating macrophages dominating with further decline in the population at 12 h. Switch in macrophage population from parasite eliminating to parasite survival occurred at 18 h. Thus, this study unveiled the importance of time-dependent cellular changes which lead to leishmaniasis.

### Sleepy macrophages appear to act as a cellular switch of macrophage phenotypes by regulating cell plasticity

Systematically, we started analyzing all the samples for their expression pattern. We checked the expression of housekeeping genes ActB and GAPDH; unexpectedly, we observed that Clusters 3 and 6 from the 6-h sample do not express housekeeping genes. To our surprise, these cells did not express IL-12b, IL-10, Ptpn6 (SHP-1), and Nfat5, although the motif *z*-score for NFAT5 was highest in these two clusters than across any other cluster in the same sample. These unique clusters were absent in other samples. Since these clusters consisted overall 28% of the population, we got intrigued about the set of genes these clusters express, transcription factors associated with these genes, pathways which are active in these clusters, and most importantly, crucial components in these clusters which are making them unique to such behavior. How the cells are managing to survive without expressing housekeeping genes is still not well understood and needs further investigation. We predict that amastigotes inside these macrophages are activating gene sets which may be conferring to their uniqueness. Even though we could identify sleepy macrophages, the mechanistic insight behind their phenotype is still not well understood. These cells did not show expression for Caspase 3 and Caspase 7, which are apoptotic markers, suggesting that sleepy macrophages were not pro-apoptotic but may be modulatory (Fig. S5).

### Principal component analysis revealed cluster 3 and its genes may be critical for sleepy macrophages

Our initial finding from PCA was that the genes elevated in Clusters 3 and 6 were downregulated in Clusters 1, 2, 4, and 5. Hence, to identify which cluster is essential, we performed the PCA and observed that 85.2% of information is retained by first three clusters. From the variable plot, we can also say that Cluster 3 is negatively correlated with other clusters with high cos2 value. As an individual PC, Cluster 3 is highly correlated with PC1 and PC2, and Cluster 6 had lesser correlation, suggesting its low contribution in the data set. We distributed the PCs based on significance of their expression and categorized 814 genes in a group of five for all clusters of which genes having *P* values of more than 0.0001 were identified, and they showed low redundancy distribution. Hence, from this analysis, we focused our studies on Cluster 3 to identify behavior of sleepy macrophages.

### Sleepy macrophages might act as transient state to prepare macrophages to combat *L. major* infection

Using GSEA we were able to analyze the defined set of genes from the 6-h sample by measuring statistically significant and concordant changes between phenotypes (sleepy macrophage and normal). GSEA was used to evaluate the relevance of numerous aberrations in gene expression and cellular transcriptional responses using single-cell ATAC gene expression data set for *Mus musculus* macrophage. Using the gene expression profiles of infected macrophages, GCT, GMT, and CLS files were prepared as input files for GSEA analysis (Tables S4 and S5). The metric used for ranking the genes in the data set was Diff_of_Classes, and the chip platform provided for the analysis was Mouse_NCBI_Gene_ID_MSigDB.v2022.1.Mm.chip. The results obtained showed that the genes belonging to Clusters 3 and 6 were positively correlated with the sleepy macrophage phenotype or state.

We analyzed the top 10 genes from the heat map by excluding pseudogenes and observed that Pcdh11x was enriched in Cluster 3, which is responsible for cell recognition and activation of PI3k/Akt signaling (37); Taf2 is critical in formation of RNA polymerase II initiation complex (38); TMEM117 primarily functions in the endoplasmic reticulum stress-mediated mitochondrial apoptotic pathway (39); Gabrg1 has a role in chloride channel activity, although we could not find its relevance in leishmaniasis or macrophages; Tox is a DNA-binding protein associated with control of the chromatin structure, majorly in the activation of T cells (40); Fastkd3 is an unusual RNA-binding protein that critically regulates mitochondrial RNA metabolism (41); the *L. major*-infected mice model has been reported to show significant reduction in parasite burden in lymph nodes, spleen, and liver when Map2 activity was enhanced *in vivo* (42); Erap2 acts as protease to cleave antigens and form peptides which are presented by MHC-I in canine leishmaniasis (43); Zfp606 has not been reported to have any role; and Vmn2r70 is predicted to have a role in enabling G protein-coupled receptor activity. From the expression set, we could infer that sleepy macrophages may have a stressful environment; they may be undergoing epigenetic and transcriptional changes to *L. major* infection.

### NFAT5 may control cellular signaling and epigenetically regulate sleepy macrophages as a response to *L. major* infection

Mef2c has been demonstrated to be associated with enrichment of classical activated macrophages that promote pro-inflammatory cytokine production in leishmaniasis (44). It was observed from patient samples that synthesis of Mef2c is associated with the NFAT5 canonical pathway, which also in turn regulates NFAT5-mediated immune response; TNF-α is the upstream regulator of MEF2C (45). In human and murine fibroblasts, Mef2c was reported to be activated at the G0/G1 phase (46). Another transcription factor which was enriched through TFTG network was Isl1, although much is not known about the role of this gene in leishmaniasis. Nonetheless, it consists of the LIM domain, which is essential for regenerating T cells in spleen in leishmaniasis (47). From the pathway enrichment analysis of all 814 genes and 51 transcription factors, we observed that NFAT5 signaling, gene transcription, MAPK signaling, and TGF-β signaling were enhanced. From this analysis, we could infer that NFAT5-mediated signaling and immune response prevail in sleepy macrophages.

### TP53-mediated cell cycle irregularities in sleepy macrophages may impact immune cell activation

From cell cycle analysis of the 6-h sample, the presence of cells in sub-G1 cells identified apoptotic cells, which corroborates with Fig. S1. Cluster 3 sleepy macrophage accounts for 19%, which was also identified through the cell cycle analysis of the 6-h sample. The reactions enriched in sleepy macrophages favored the G1 phase of the cell cycle (Fig. 9E) Therefore, it may be possible that sleepy macrophages are in the G0/G1 phase (Fig. 9A). As p53 expression was pertinent in some sleepy macrophages, they may lead to G1 cell cycle arrest as it has been reported in leishmaniasis (48) at the G1 phase (49). H2-D is involved in CD8^+^ T-cell activation and CD4^+^ Th1 response in leishmaniasis (50, 51). H2-D1 downregulation was an intimation of decreased or irregular antigen processing and presentation and challenged the abilities of macrophages to act as antigen presenting cells when in sleepy state. These results indicated that parasites may regulate the MHC-II expression in hosts as a shielding mechanism to later grow and proliferate, and this may be strongly associated with the transcription factor and their target genes, which are associated with the cell cycle regulation and chromatin remodeling.

### Expression of IL-10, IL-12, NFAT5, and SHP-1 reveals similar expression trend at protein level as identified from sc-ATAC sequencing expression analysis

From our previously reported finding (28), we observed the expression change of IL-10, IL-12, and SHP-1 with different time points of infection. Correspondingly, we observed the same trend in peritoneal macrophages with the same time points of infection. Perhaps, it may be possible that RAW264.7 cells and peritoneal macrophages may respond to infection in an identical way at the same time points. This may propose a possibility of sleepy macrophage depicting behavior in peritoneal macrophages as derivation of RAW264.7 cells was from pristane-elicited peritoneal cells of Balb/c mice (52). RAW264.7 cells also show parallel immune response to primary murine bone-derived macrophages through TLR 2 and TLR 4 signaling (53), which is a key to CL infection from our previously reported mathematical models (26).

### Conclusion

Our findings identified sleepy macrophages which possess a state adaptation to *L. major* infection. These cells boast pro-inflammatory cytokine expression by promoting chromatin remodeling and RNA regulation through transcription factors. One such transcription factor which we have highlighted from our findings is NFAT5 that dictates IL-10 and IL-12 reciprocal regulation in *L. major* infection. Often NFAT5 gets downregulated by *L. major*-induced SHP-1-mediated dephosphorylation of its auxillary export domain. It may be upright to target NFAT5 so as to direct adequate parasite elimination response. We presume that inhibition of SHP-1 may prevent NFAT5 inhibition and suppress parasite survival. Our previous work has already underlined the proposition in which peptides will be used to inhibit SHP-1. In the future, we will inspect the efficacy of peptides on macrophage populations in *L. major* infection.

## Data Availability

This Single Cell ATAC sequencing project raw files have been deposited in the National Center for Biotechnology Information Sequence Read Archive under accession number PRJNA1061857. The deposited sequence may be found in https://www.ncbi.nlm.nih.gov/bioproject/PRJNA1061857. Any data that support the findings of this study beyond what is included in the supplemental information are available from the corresponding author upon request.

## SUPPLEMENTAL MATERIAL

Supplemental figures spectrum.03478-23-s0001.docx Figures S1 to S5.

Supplemental tables spectrum.03478-23-s0002.xlsx Tables S1 to S8.

ASM does not own the copyrights to Supplemental Material that may be linked to, or accessed through, an article. The authors have granted ASM a non-exclusive, world-wide license to publish the Supplemental Material files. Please contact the corresponding author directly for reuse.
