# Supplementary material for: Single-cell ATAC sequencing identifies sleepy macrophages during reciprocity of cytokines in L. major infection
Source: Microbiol Spectr. 2024 Feb 1;12(3):e03478-23. doi: 10.1128/spectrum.03478-23 (PMC10913457; doi:10.1128/spectrum.03478-23)
Supplement: Supplemental figures — Figures S1 to S5. [file spectrum.03478-23-s0001.docx]

Supplementary file


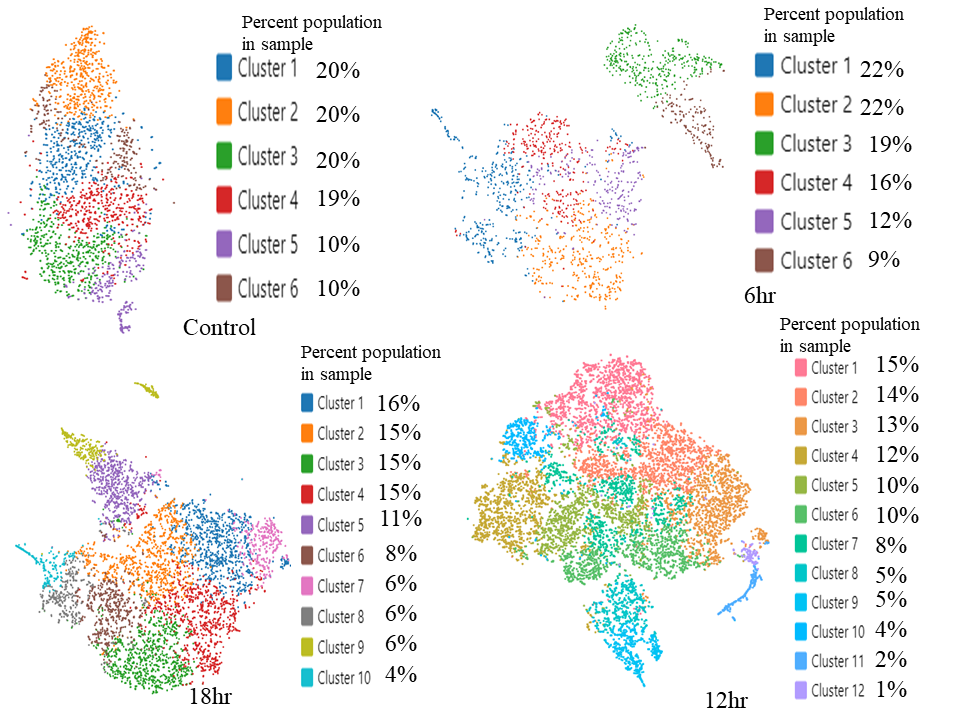


Figure S1: Percent population of all clusters from individual sample represented as t-SNE plot


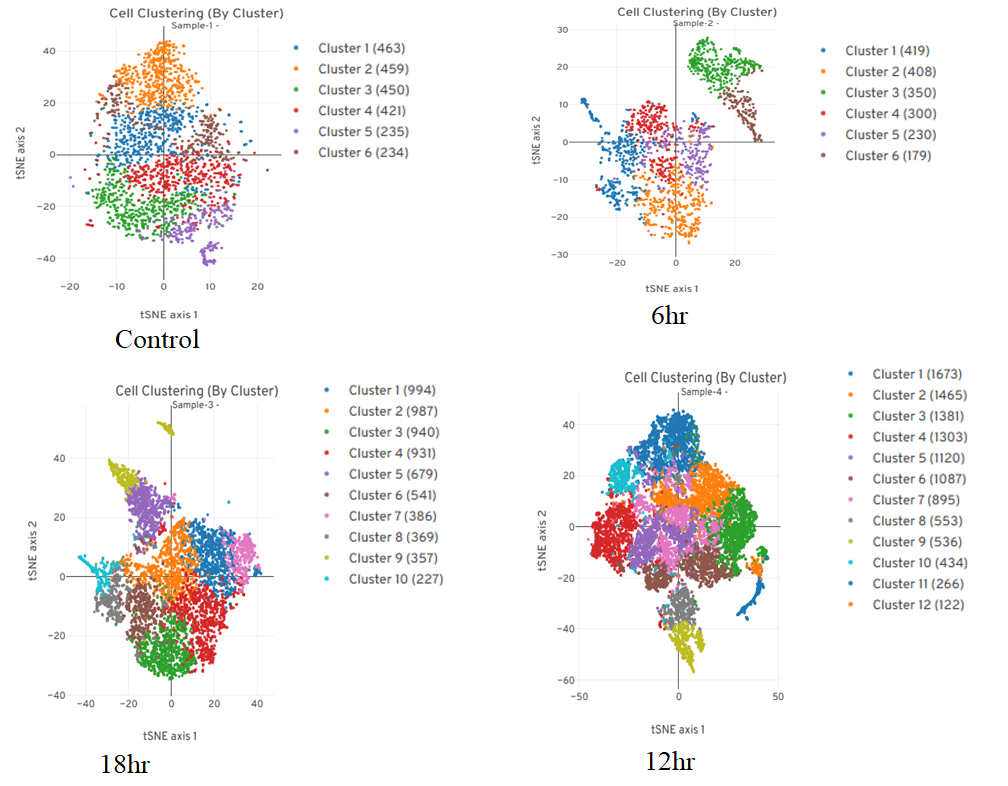


Figure S2: Cell number of each cluster in the individual sample represented in t-SNE plot


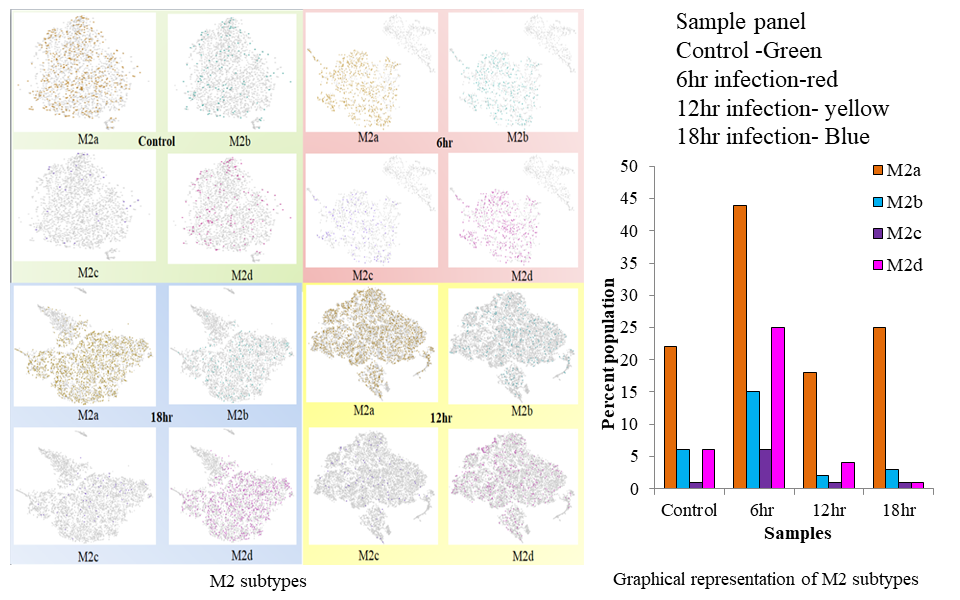


Figure S3: M2 macrophage subtype population (M2a, M2b, M2c and M2d) post infection from control, 6hr, 12hr and 18hr infected samples.


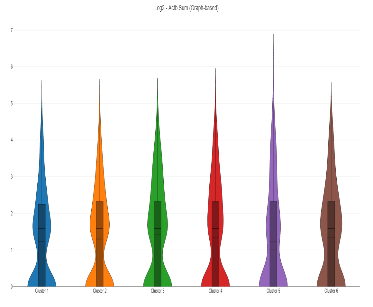

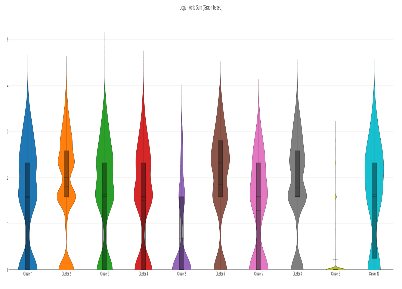

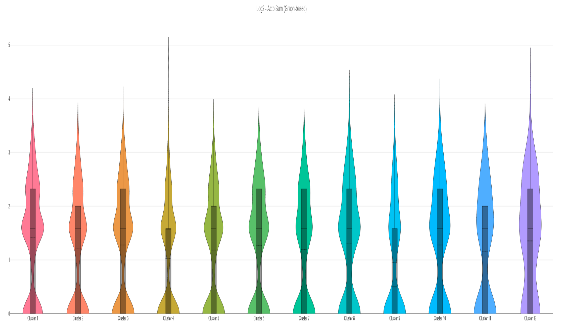


Log2 fold change of Gapdh

Log2 fold change of ActB

18hr infected

12hr infected

Control


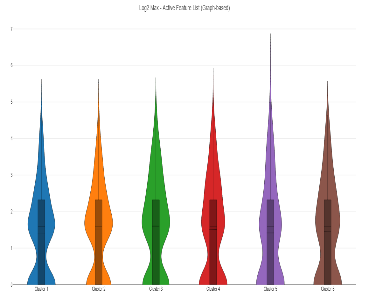

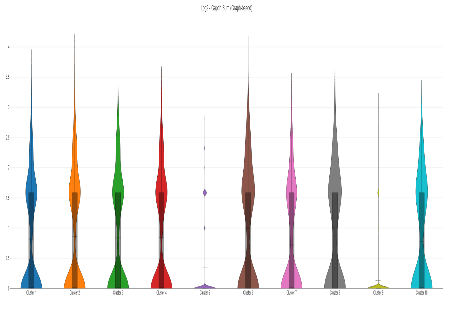

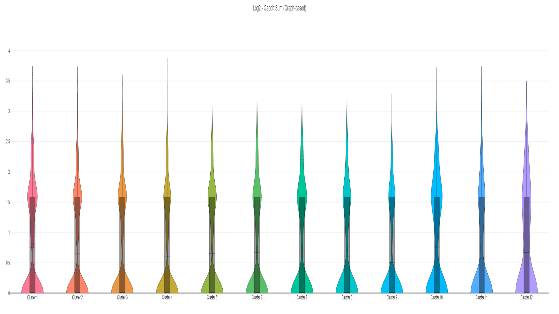


Log2 fold change of IL-10


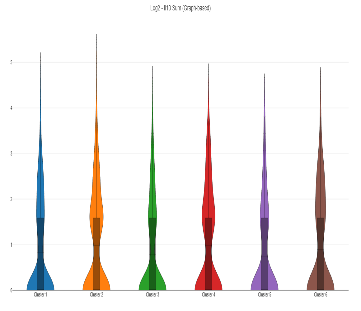

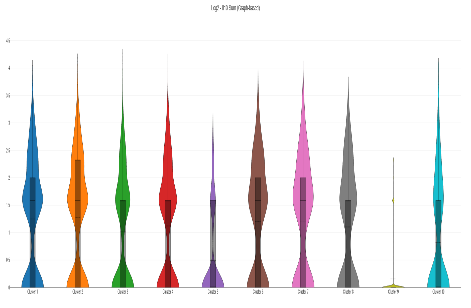

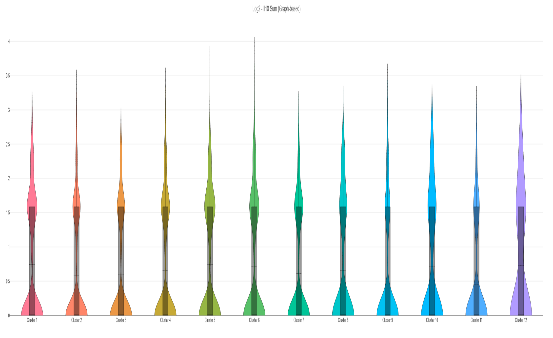


Log2 fold change of IL-12b


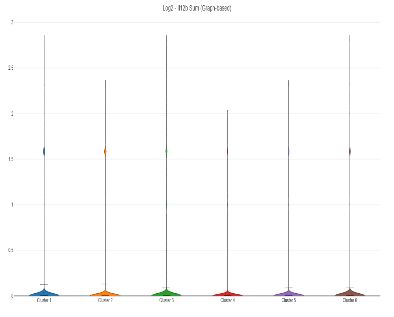

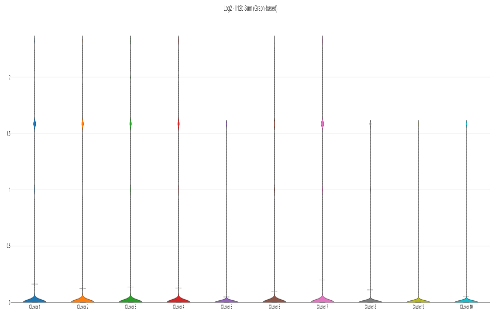

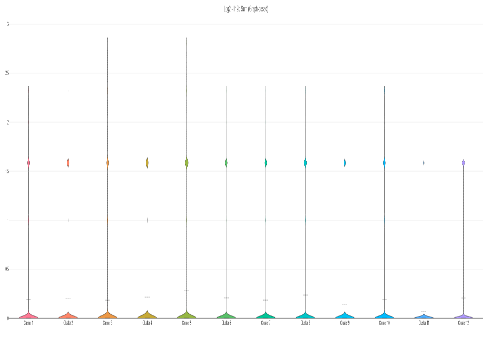


Motif z-score of Nfat5


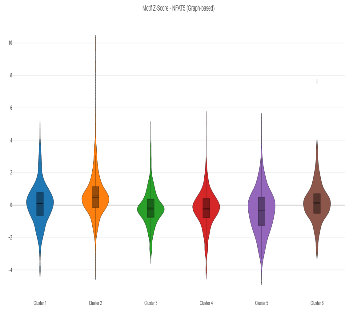

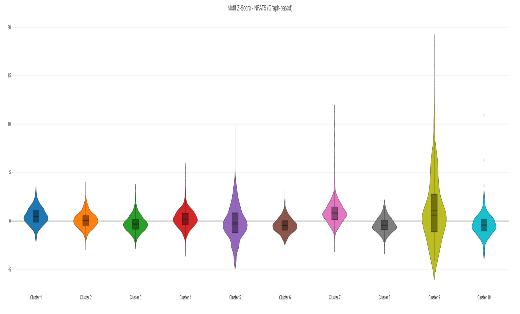

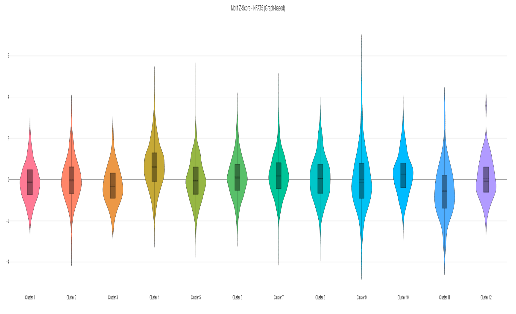


Log2 fold change of Ptpn6


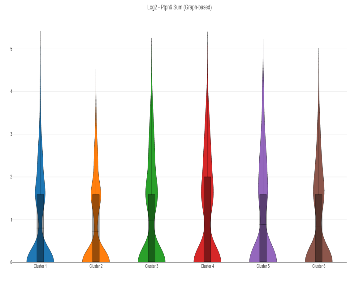

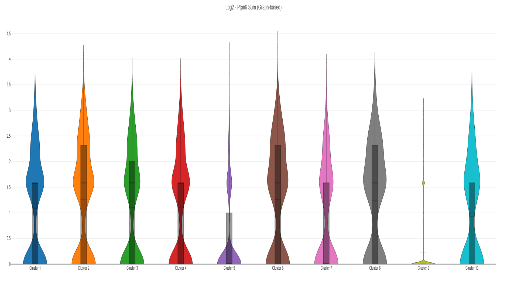

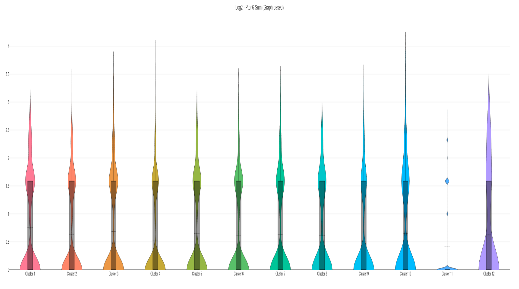


Figure S4: Violin plot of gene expression of ActB, Gapdh, IL10, IL12b, Ptpn6, and motif z-score of NFAT5 in control, 12hr *L.major* infected and 18hr *L.major* infected samples.


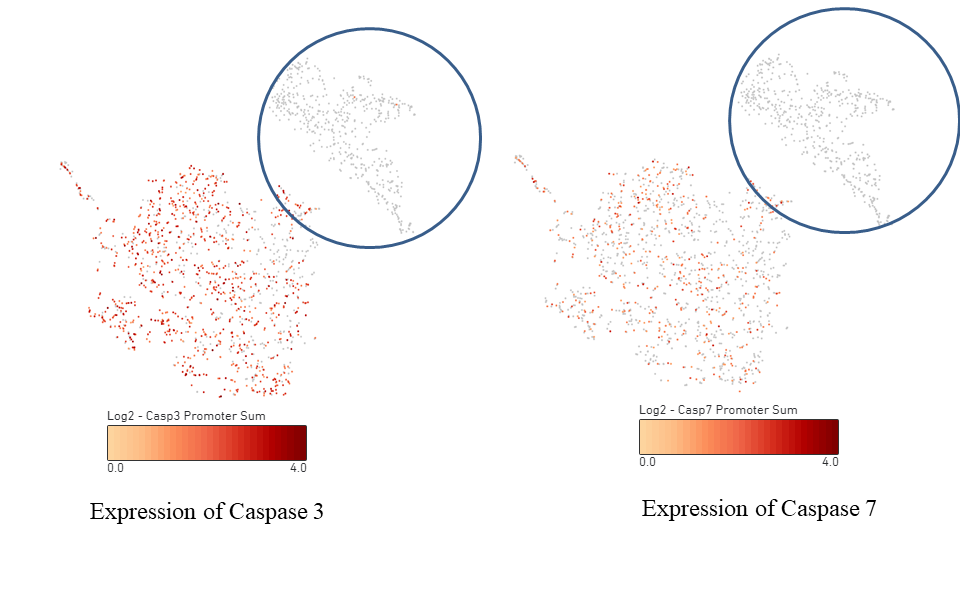


Figure S5: Gene expression of apoptotic markers showing sleepy macrophages might not be pro-apoptotic
